# Genetic polymorphisms of the sortase A gene and social-behavioural factors associated with caries in children: a case–control study

**DOI:** 10.1186/s12903-015-0039-1

**Published:** 2015-05-02

**Authors:** Li Xia Yu, Ye Tao, Rong Min Qiu, Yan Zhou, Qing Hui Zhi, Huan Cai Lin

**Affiliations:** Department of Preventive Dentistry, Guanghua School of Stomatology, Sun Yat-Sen University, 56 Ling Yuan Road West, Guangzhou, China; Guangdong Provincial Key Laboratory of Stomatology, Sun Yat-Sen University, Guangzhou, China

**Keywords:** Caries, Gene polymorphisms, *srtA*, *Streptococcus mutans*

## Abstract

**Background:**

*Streptococcus mutans* (*S. mutans*) is the primary etiological agent of dental caries. Sortase is a transpeptidase that anchors several surface proteins to the *S. mutans* cell wall and has been shown to play a major role in cariogenicity. The purpose of this study was to explore the genetic polymorphisms of the sortase gene (*srtA*) and the social-behavioural factors associated with dental caries in children with *S. mutans*.

**Methods:**

In this case–control study, 121 *S. mutans* strains were separately selected from caries-free children and high-severity caries children for sequencing of the *srtA* gene. Social and behavioural data were collected by self-administered questionnaires. Genomic DNA was extracted from *S. mutans* strains and amplified by PCR to obtain the *srtA* gene. The purified PCR products were sequenced and analysed for mutations with ABI Variant Reporter software. The distribution of missense mutations and the mean of social-behavioural factors were compared between the groups. A multiple logistic regression model was used to control for confounding factors.

**Results:**

The mutation frequencies at loci 168 (*P* = 0.023) and 470 (*P* = 0.032) were significantly different between the groups. The best-fitting model showed that greater age, high frequencies of solid sugar consumption, prolonged breastfeeding, a high proportion of visible plaque, and *S. mutans* with a T at locus 168 of the *srtA* gene were associated with high-severity caries in children (*P* < 0.05). Children carrying a G at locus 168 of *S. mutans* had a decreased risk for high-severity caries (OR = 0.32, 95% CI = 0.12–0.86) compared with those carrying a T.

**Conclusions:**

The present study suggested that the locus 168 missense mutation of the *srtA* gene may correlate with caries susceptibility in children *with S. mutans*. In addition, age, duration of breastfeeding, solid sugar consumption, and poor oral hygiene contributed to this complex disease.

## Background

Dental caries is the localised destruction of dental hard tissues by acidic by-products from bacterial fermentation of dietary carbohydrates [1]. Oral microorganisms, dietary habits and host susceptibility interact in the initiation and development of dental caries [2]. In general, socio-demographic variables, development characteristics, general upbringing, oral health related behaviour, oral hygiene and bacteria are known risk factors for the development of dental caries in children [3]. It is a common worldwide disease affecting children’s health and well-being [2,4,5], especially in China. The Third National Oral Health Survey in China showed that the prevalence of childhood caries in the 5-year-old age group was as high as 66% and that the mean number of decayed, missing and filled teeth (dmft) was 3.5 [6].

*Streptococcus mutans* (*S. mutans*) is the primary etiological agent of dental caries [7]. Although the association between *S. mutans* and dental caries seems convincing, some children with *S. mutans* do not manifest the disease, suggesting that *S. mutans* may vary in its ability to initiate caries. One important characteristic of *S. mutans* in the development of dental caries is its ability to adhere to the tooth surface. Pac is one of the cell wall-anchored surface proteins identified in *S. mutans*, and it is responsible for mediating the adherence of *S. mutans* to tooth surfaces [8]. Sortase A (SrtA), coded by the gene *srtA*, has been shown to be a membrane-localized transpeptidase that covalently links protein Pac with a sorting signal to the cell wall and possesses important adherent functions that have been associated with cariogenicity [9,10]. *S. mutans* with a mutated *srtA* gene was shown to result in a marked reduction in the adhesion potential of *S. mutans* and the frequency of dental caries [11]. Because an important function of *srtA* is the adherence of *S. mutans* to the tooth surface, we hypothesized that the *srtA* gene of *S. mutans* might possess genetic polymorphisms related to different caries conditions.

Considering the complex etiology of dental caries, the virulence and colonization of *S. mutans* can be modulated by behavioural, social and environmental factors [12,13]. In this study, we aimed to explore the genetic polymorphisms of the *srtA* gene and the social-behavioural factors associated with dental caries in children with *S. mutans*.

## Methods

### Calculation of study sample size

A case–control group design was applied in this study. According to the study design, the formulas used to calculate the sequencing sample capacities are shown below [14].

Formula 1: $$ N=\frac{N^{\hbox{'}}}{4}{\left(1+\sqrt{1+\frac{4}{N^{\hbox{'}}\delta }}\right)}^2 $$

Formula 2: $$ {N}^{\hbox{'}}=\frac{{\left[{Z}_{\alpha}\sqrt{\left(1+1/C\right){\pi}_c\left(1-{\pi}_c\right)}+{Z}_{\beta}\sqrt{\pi_2\left(1-{\pi}_2\right)+{\pi}_1\left(1-{\pi}_1\right)/C}\right]}^2}{{\left({\pi}_2-{\pi}_1\right)}^2} $$

Formula 3: $$ {\pi}_c=\frac{\pi_2+{\pi}_1}{2} $$

Formula 4: *δ* = |*π*_1_ − *π*_2_|

The number of children was set to be equal in the case and control groups. Thus, the value of *C* was 1.0. The value of α was set at 0.05, and the value of β was set at 0.15. The values of *π*_1_ and *π*_2_ referred to the predicted missense mutation rates of *srtA* in the control and case groups in the present study, respectively. Based on the rates of each missense mutation locus in the caries-free and caries-active groups of our previous work [15], the largest sample size was required when *π*_1_ =0.6 and *π*_2_ =0.4, respectively. It was therefore calculated that the sample size should be 121 children for each group. All statistical tests were two-sided.

Because this study aimed primarily to explore connections between missense mutations of *srtA* and the severity of caries in the children with *S. mutans*, only the children who carried *S. mutans* were analysed. To satisfy the required sample size, we calculated the number of children that was required for the epidemiological survey. The prevalence rate of caries (68%) and non-caries (32%) in young children [16], along with the prevalence rate of *S. mutans* in the caries-free group (37.5%) and the caries-active group (75%), were considered [17]. The minimum number of children required to investigate was therefore calculated to be 1,009.

### Field investigation

An epidemiological survey was carried out in Huadu District of Guangzhou in South China from October 2012 to June 2013. The study protocol was approved by the Ethics Committee of Guanghua School of Stomatology, Sun Yat-sen University (ERC-[2012]-13). The Huadu District is a new urban district that consists of four streets and six towns. There were 114 nursery schools in this district. A random cluster sampling technique was employed to select 19 schools according to the number of children that we needed to recruit. Only those children who were aged 36–47 months old, had lived in the district for more than six months, reported no systematic illness, and reported no antibiotic intake for at least the preceding one month were included in the study. All of the participating schools were informed of and consented to the study. After written parental consent was obtained, all eligible 3-year-old children in the participating schools were included in the study.

Caries development, enamel hypoplasia and visible plaque accumulation were determined by a single dentist (L.X. Yu). CPI probes, disposable mouth mirrors, and intra-oral LED light sources were used for the examinations. The status of the dental caries was recorded according to the World Health Organization criteria using dmft indexes [18]. In short, the presence of dental caries was recorded when there was an obvious lesion in a pit or fissure or on smooth surface of a tooth. A detectable softened wall or undermined enamel was also recorded as dental caries. Enamel hypoplasia was recorded using the criteria recommended by the Fédération Dentaire Internationale (FDI) for general epidemiological surveys [19]. Enamel hypoplasia included three types of defects: pits, grooves or missing enamel. Oral hygiene was assessed using the Visible Plaque Index (VPI) [20]. Four sites of distal, midmost and mesial of buccal surfaces and the midmost of the lingual surface of each tooth were examined to record the VPI. The percentage of the examined sites with visible plaque was calculated. Approximately 10% of the subjects were re-examined to assess the intra-examiner reliability. Pooled samples of dental plaque from each child were collected with sterile cotton swabs from the buccal surfaces of maxillary teeth. The samples were dispersed in a sterile fluid thioglycolate (FT) medium and taken to the laboratory on ice within 4 h of collection.

Data were collected using a self-administered questionnaire that was administered to the caregivers. The questionnaire consisted of four parts: socio-demographic characteristics (e.g., age and sex of children, occupation and education level of parents), developmental characteristics (e.g., gestational age, mode of delivery, weight at birth and enamel hypoplasia), general upbringing history (e.g., bottle-feeding experience and duration of breastfeeding) and oral health behaviour (e.g., solid sugar consumption, frequency of tooth brushing and use of toothpaste).

### Isolation of *S. mutans*

Plaque samples were mixed and sonicated for 30 s and were dispersed to obtain a dilution series to 10^−3^ dilutions. For each sample, 50 μl of the diluent was plated onto Mitis-Salivarius-Bacitracin (MSB) agar, supplemented with 20% sucrose and 0.2 units/ml bacitracin and incubated anaerobically (85% N_2_, 5% CO_2_, and 10% H_2_) at 37°C for 3 d [21]. We randomly selected two colonies from each child according to the colony morphology and tested the colonies for their ability to ferment mannitol, sorbitol, raffinose, melibiose, and aesculin and for their ability to hydrolyse arginine [22]. The identified bacterial strains were subsequently streaked onto MSB agar and preserved in 50% glycerol at −80°C before use.

### Defining the case and control groups

To explore and compare the genetic polymorphisms in the *srtA* gene of *S. mutans*, children with distinct caries experiences were taken into consideration. A total of 121 caries-free children with *S. mutans* were randomly selected as the caries-free group, and 121 children with dmft ≥6 who were *S. mutans-*positive were selected to form the high-severity caries group. The dmft score of the high-severity group was in accordance with the category used in a previous study [23].

### Extraction of chromosomal DNA

*S. mutans* strains were grown in 2 ml of brain-heart infusion broth and incubated at 37°C under anaerobic conditions for 18 h. Cells centrifuged from the BHI cultures were suspended in 5% Chelex100, treated with 10 μl of 20 mg/ml proteinase K at 37°C for 1 min, and then digested at 56°C for 1 h, followed by boiling for 10 min. The tubes were frozen on ice for 3 min, and the suspension was centrifuged at 12,000 rpm for 10 min. The supernatant was obtained for PCR. The quality and quantity of DNA samples were measured with a UV spectrophotometer at 260 nm and 280 nm. All DNA was stored at −20°C before further analysis.

### Amplification and sequencing of the *srtA* gene

The PCR primers designed by ABI Primer Designer V3.0 that were used to amplify the UA159 *srtA* gene are listed in Table 1. A 1,035 bp DNA fragment carrying the *srtA* gene was amplified from *S. mutans* strains. Due to the limitation of the length of sequencing reads, we amplified and sequenced the gene in three fragments that contained overlapping sections.

**Table 1:** **PCR primers used for detection of the** ***srtA*** **gene in** ***S. mutans***

**Primer**	**Sequence (5’-3’)**	**Product size (bp)**
Pair1-F	GACGTTTGGCAACTGGTGTG	557
Pair1-R	CCAAGCAATTAGGGCATTTC	
Pair2-F	CAATGAAAAAAGAACGTCAATCTA	448
Pair2-R	TGTGAAGATCCGGTCATACCA	
Pair3-F	CGGAATTGCCATTCCAGACT	721
Pair3-R	TCCGAAACTATCAAAGCAACAT	

The PCR reaction was carried out in a 25 μl reaction volume. The components in the PCR reaction (final conc.) were 2.5 μl of 10 × PCR buffer, 0.2 mM of dNTP mix, 1.5 mM MgCl_2_, 0.2 μM each primer, 100–400 ng of genomic DNA as template, and 2U of Platinum® Taq DNA Polymerase (Invitrogen, CA, USA). The temperate was preheated at 95°C 5 min. The PCR cycle was as follows: denaturation at 95°C for 30 s, annealing at 60°C for 30 s, and elongation at 72°C for 50 s. A total of 50 cycles were performed, followed by a final elongation step at 72°C for 5 min. Five microliters of each amplified products was analysed by electrophoresis on a 1.5% agarose gel. The PCR products were purified using a QIAquick Gel Extraction Kit (QIAgen, Hilden, Germany). Ultimately, the products were sequenced by the Shanghai Life Technologies Biotechnology Company (Life Technologies, Shanghai, China). Variant Reporter software was used to analyse the sequencing results, and the *srtA* sequence of *S. mutans* UA159 was selected as a reference sequence.

### Statistical analysis

Data analysis was carried out using the SPSS 16.0 software. Categorical and continuous variables were compared using a Chi-square test and an independent samples *t* test, respectively. Bivariate and multivariate logistic analyses were used to calculate odds ratios (ORs) with their corresponding 95% confidence intervals (CIs) and identify the factors associated with high-severity caries. Caries status was treated as the dependent variable (0 = caries free group, 1 = high-severity caries group). Independent variables were those factors that may have influenced caries status. Those independent variables with *P* < 0.2, based on a bivariate logistic analysis, were tested further in a multiple logistic regression model. A *P* value <0.05 for all two-sided statistical tests was considered significant.

## Results

The statistical analysis of the socioeconomic demographic characteristics and developmental factors are shown in Table 2. In social indicators, we found significantly different distributions in the ages (in months) between the groups (*P* < 0.001). Among the variables representing children’s oral health behaviour (Table 3), the duration of breastfeeding (*P* = 0.09), frequency of solid sugar consumption (*P* < 0.01) and the proportion of VPI (*P* < 0.01) were all significantly associated with caries risk.

**Table 2:** **Bivariate analysis of demographic, socio-economic and development characteristics in relation to caries status**

**Variables**	**Controls (n = 121)**		**Cases (n = 121)**		***x*** ^**2**^	P-value*	**COR (95% CI)**	**P-value**
	**n (%)**		**n (%)**					
**Part1 Demographic and socio-economic characteristics**								
**Sex**					1.345	0.246		
Males^†^	59	(48.8)	69	(57.0)				
Females	62	(51.2)	52	(43.0)			0.72(0.43-1.19)	0.198
**Mother’s schooling**					1.369	0.242		
≥12 years^†^	74	(61.2)	65	(53.7)				
<12 years	47	(38.8)	56	(46.3)			0.74(0.44-1.23)	0.242
**Father’s schooling**					3.615	0.057		
≥12 years^†^	87	(71.9)	73	(60.3)				
<12 years	34	(28.1)	48	(39.7)			0.59(0.35-1.02)	0.058
**Mother’s occupation**					1.813	0.404		0.413
Employer⁄Professional^†^	8	(6.6)	14	(11.6)				
Employee⁄Non-professional	88	(72.7)	84	(69.4)			0.55(0.22-1.37)	0.196
Unemployed	25	(20.7)	23	(19.0)			0.53(0.19-1.48)	0.224
**Father’s occupation**					3.503	0.173		0.181
Employer⁄Professional^†^	22	(18.2)	29	(24.0)				
Employee⁄Non-professional	92	(76.0)	90	(74.4)			0.74(0.40-1.39)	0.350
Unemployed	7	(5.8)	2	(1.7)			0.22(0.04-1.15)	0.072
	Mean(SD)		Mean(SD)		*t* test			
**Age (months)**	41.6	(2.9)	43.4	(3.6)	−4.285	<0.001**	1.18(1.09-1.28)	<0.001
**Mother’s age at child’s birth**	27.1	(3.8)	26.4	(3.8)	1.517	0.131**	0.95(0.89-1.02)	0.132
**Part 2 Development characteristics**								
**Gestational age**					0.066	0.797		
≥37 weeks^†^	58	(47.9)	60	(49.6)				
<37 weeks	63	(52.1)	61	(50.4)			0.94(0.57-1.55)	0.797
**Mode of delivery**					0.281	0.596		
Vaginal birth^†^	73	(60.3)	77	(63.6)				
Caesarean birth	48	(39.7)	44	(36.4)			0.87(0.52-1.46)	0.596
**Weight at birth**					2.381	0.123		
≥2500 g^†^	118	(97.5)	113	(93.4)				
<2500 g	3	(2.5)	8	(6.6)			2.79(0.72-10.76)	0.138
**Enamel hypoplasia**					—	—		
No†	121	(100.0)	120	(99.2)				
Yes	0	(0.0)	1	(0.8)			—	—

*Chi-square test, **Independent samples *t* test.

COR (crude odds ratio), CI (confidence interval), ^†^Reference Category.

**Table 3:** **Bivariate analysis of general upbringing and oral health behaviour in relation to caries status**

**Variables**	**Controls (n = 121)**		**Cases (n = 121)**		***x*** ^**2**^	P-value*	**COR (95% CI)**	**P-value**
	**n (%)**		**n (%)**					
**Part 1 General upbringing between 0–3 years**								
**Bottle-feeding experience**					0.764	0.382		
Yes^†^	22	(18.2)	17	(14.0)				
No	99	(81.8)	104	(86.0)			1.36(0.68-2.71)	0.383
**Duration of breastfeeding**					9.382	0.009		0.012
Never breastfed^†^	22	(18.2)	9	(7.4)				
<1 year	84	(69.4)	84	(69.4)			2.44(1.06-5.62)	0.035
≥1 year	15	(12.4)	28	(23.1)			4.56(1.68-12.37)	0.003
**Part 2 Oral health behaviour at age 3**								
**Solid sugar consumption**					19.492	<0.001		
<1 time per day^†^	86	(71.1)	52	(43.0)				
≥1 time per day	35	(28.9)	69	(57.0)			3.26(1.91-5.56)	<0.001
**Frequency of tooth brushing**					0.154	0.695		
≥1 time per day^†^	73	(60.3)	70	(57.9)				
<1 time per day	48	(39.7)	51	(42.1)			1.11(0.66-1.85)	0.695
**Use of toothpaste**					0.000	1.000		1.000
Always^†^	70	(57.9)	70	(57.9)				
Sometimes	29	(24.0)	29	(24.0)			1.00(0.54-1.84)	1.000
Never	22	(18.2)	22	(18.2)			1.00(0.51-1.97)	1.000
	Mean(SD)		Mean(SD)		*t* test			
**Visible plaque index (%)**	46.2	(20.9)	75.7	(15.8)	−12.386	<0.001**	1.08(1.06-1.10)	<0.001

*Chi-square test, **Independent samples *t* test.

COR (crude odds ratio), CI (confidence interval), ^†^Reference Category.

**Figure 1:**
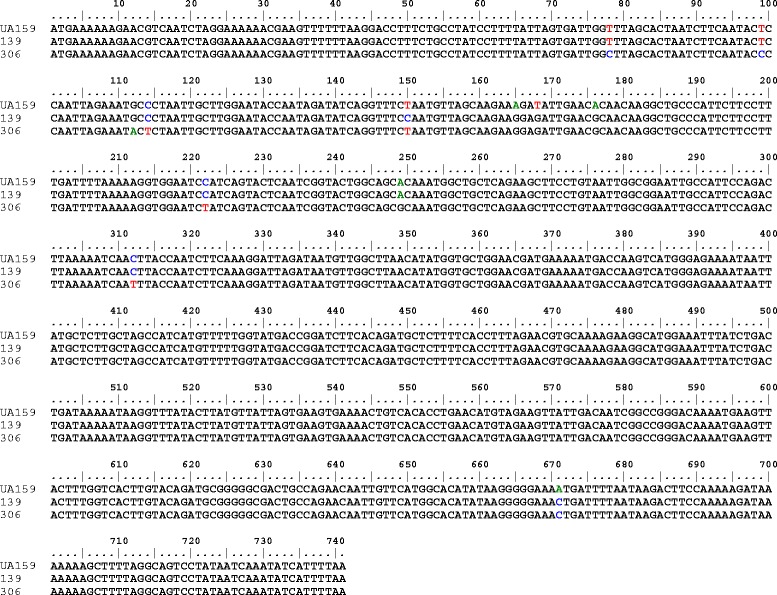
Point mutations in clinical isolates. Detailed legend: The No. 306 clinical isolate (caries-free group) has point mutations at 78, 99, 112, 114, 165, 168, 176, 222, 249, 312, and 671 locus bases. The No. 139 clinical isolate (high-severity caries group) had a point mutation at 78, 150, 165, 168, 176, 671 locus bases.

In comparing the *srtA* sequences of the clinical strains with *S. mutans* UA159, a total of 38 single nucleotide substitutions were found, including 21 silent mutation sites and 17 missense mutation sites (Figure 1). The caries-free group was found to have 19 silent mutation sites and 11 missense mutation sites, whereas the high-severity caries group was found to have 20 silent mutation sites and 14 missense mutation sites. Only ten strains were identical to strain UA159; of these, five were from the caries-free group, and five were from the high-severity caries group. None of the *srtA* genes in the sequences had a base insertion or deletion.

Silent mutation sites in the clinical strains were identified at positions 48, 78, 85, 99, 138, 150, 162, 165, 183, 186, 222, 237, 249, 261, 312, 357, 582, 615, 636, 669, and 717.

Missense mutation sites in the clinical strains were identified at positions 23, 34, 36, 47, 100, 112, 114, 168, 176, 256, 298, 382, 470, 548, 584, 671, and 706. The amino acid transversions due to missense mutations are displayed in Table 4. Here, we only show the alterations of amino acids due to missense mutations because these changes could affect the activity of sortase A.

**Table 4:** **Transversion of amino acids due to missense mutations according to codons**

**Base site**	**UA159**		**Clinal strains**	
	**Codon**	**Amino acid**	**Codon**	**Amino acid**
23	AGG	Arginine	AAG	Lysine
34	AGT	Serine	GGC/GGT	Glycine
36	AGT	Serine	GGC/GGT	Glycine
47	ACC	Threonine	ATC	Isoleucine
100	CCA	Proline	TCA	Serine
112	GCC	Alanine	ACC/ACT	Threonine
114	GCC	Alanine	ACT	Threonine
168	GAT	Asparaginic acid	GAG	Glutamic acid
176	CAC	Histidine	CGC	Arginine
256	GCT	Alanine	TCT	Serine
298	GAC	Asparaginic acid	AAC	Asparagine
382	GTC	Valine	ATC	Isoleucine
470	CGT	Arginine	CAT	Histidine
548	GTC	Valine	GCC	Alanine
584	CCG	Proline	CTG	Leucine
671	AAT	Asparagine	ACT	Threonine
706	GCT	Alanine	ACT	Threonine

The distribution frequencies of the missense mutation sites are listed in Table 5. There was a significant difference in the mutation frequency at locus 168 (*P* = 0.023); the frequency of mutations at this site was significantly higher in the caries-free group than in the high-severity caries group. Moreover, strains with the locus 470 polymorphism exhibited a significantly higher rate in the high-severity caries group compared with the caries-free group (*P* = 0.032).

**Table 5:** **Bivariate analysis of the missense mutation rates in relation to caries status**

**Missense mutation**	**Controls (n = 121)**		**Cases (n = 121)**		***x*** ^**2**^	P-value*	**COR (95% CI)**	**P-value**
	**n (%)**		**n (%)**					
23 G → A^†^	5	(4.1)	12	(9.9)	3.100	0.078	2.55(0.87-7.49)	0.087
34 A → G	7	(5.8)	10	(8.3)	0.569	0.450	0.68(0.25-1.85)	0.453
36 T → C	6	(5.0)	11	(9.1)	1.582	0.209	1.92(0.69-5.36)	0.215
47 C → T	8	(6.6)	11	(9.1)	0.514	0.473	1.41(0.55-3.64)	0.475
100 C → T	0	(0.0)	1	(0.8)	—	1.000**	—	1.000
112 G → A	71	(58.7)	71	(58.7)	0.000	1.000	1.00(0.60-1.67)	1.000
114 C → T	65	(53.7)	56	(46.3)	1.339	0.247	0.74(0.45-1.23)	0.248
168 T → G	26	(21.5)	13	(10.7)	5.166	0.023	0.44(0.21-0.90)	0.025
176 A → G	63	(52.1)	68	(56.2)	0.416	0.519	1.18(0.71-1.96)	0.519
256 G → T	1	(0.8)	0	(0.0)	—	1.000**	—	1.000
298 G → A	1	(0.8)	0	(0.0)	—	1.000**	—	1.000
382 G → A	1	(0.8)	0	(0.0)	—	1.000**	—	1.000
470 G → A	7	(5.8)	17	(14.0)	4.625	0.032	2.66(1.06-6.68)	0.037
548 T → C	0	(0.0)	1	(0.8)	—	1.000**	—	1.000
584 C → T	0	(0.0)	1	(0.8)	—	1.000**	—	1.000
671 A → C	115	(95.0)	116	(95.9)	0.095	0.758	0.83(0.25-2.78)	0.758
706 G → A	0	(0.0)	1	(0.8)	—	1.000**	—	1.000

*Chi-square test. **Fisher’s exact test.

^†^G → A, G represents the 23 locus base in UA159, A represents the 23 locus base in the clinical strains.

COR (crude odds ratio), CI (confidence interval).

To control for confounding factors, multiple logistic regression analyses were performed, and the results (Table 6) showed that greater age (*P* = 0.027), high frequencies of solid sugar consumption (*P* < 0.001), prolonged breastfeeding (*P* = 0.028), a high proportion of visible plaque (*P* < 0.001), and *S. mutans* strains with a T at locus 168 of the *srtA* gene (*P* = 0.023) were significantly associated with high-severity caries in children. A lower risk of high-severity caries (AOR = 0.32, 95% CI = 0.12-0.86) was found in children who carried *S. mutans* strains with a G at locus 168 of the *srtA* gene in comparison to a T. However, after controlling for confounding factors, the mutation at locus 470 was excluded from the model.

**Table 6:** **Summary of the multiple logistic regression results**

**Variables**	**B** ^**‡**^	**SE**	**P**	**AOR**	**95% CI for AOR**	
					**Lower**	**Upper**
Mutations at locus 168						
No^†^						
Yes	−1.156	0.510	0.023	0.32	0.12	0.86
Duration of breastfeeding			0.028			
Never breastfed^†^						
<1 year	1.273	0.651	0.050	3.57	1.00	12.79
≥1 year	2.058	0.772	0.008	7.83	1.73	35.54
Solid sugar consumption						
<1 time per day^†^						
≥1 time per day	1.911	0.427	<0.001	6.76	2.93	15.61
Age (months)	0.131	0.059	0.027	1.14	1.02	1.28
Visible Plaque Index (%)	0.090	0.012	<0.001	1.09	1.07	1.12
Constant	−16.110	3.263	<0.001	0.00		

^†^Reference Category, ^‡^B: regression coefficient.

SE (standard error), AOR (adjusted odds ratio), CI (confidence interval).

## Discussion

The present study compared the genetic diversity in the *srtA* gene of *S. mutans* strains isolated from caries-free and high-severity caries children. Bivariate analysis of the present work showed that the mutation at locus 168 was overrepresented in the caries-free group and that the mutation at locus 470 was overrepresented in the high-severity caries group. Importantly, in our previous work [15], the mutation rate of locus 168 was higher in the caries-free group than in the caries-active group, and the mutation rate of locus 470 was higher in the caries-active group than in the caries-free group. The distributions in the mutation frequency of these two loci in the two groups were in accordance with the results of the present study.

Because bivariate analysis cannot exclude the confounding effect of other risk factors on caries in children, a multiple logistic regression was used. After adjusting for children’s socio-demographics, developmental characteristics, general upbringing and oral health-related behavioural factors, the association between the *srtA* locus 470 polymorphisms and high-severity caries was abolished, but the association between the *srtA* locus 168 polymorphisms and high-severity caries remained. This result demonstrated that the *S. mutans* strains with a point mutation at locus 168 in *srtA* may have a lower risk for high-severity caries. It has been reported that *S. mutans* Ingbritt and NG5 preserved in the laboratory both have low cariogenicity due to the mutations in the *srtA* gene. Sequence analysis revealed that strain Ingbritt had an 11-nt deletion in the gene compared with strain GS5 [24] and strain NG5 had a single base substitution from G to T at the codon GAA, which codes for glutamic acid in NG8 [11]. Without a functional sortase, *S. mutans* strains were not able to perform several cell surface-related activities, including saliva-mediated adherence and aggregation [11,24]. Hence, we speculate that the point mutation at locus 168 may lead to a lower-activity sortase, which could affect the virulence of adherence and aggregation of *S. mutans*.

In this study, we aligned the nucleotide sequences of the *srtA* gene from clinical strains with those of strain UA159. The comparison showed that none of the clinical strains had base deletions, as observed for *S. mutans* Ingbritt. Although a single base substitution was a common occurrence in *S. mutans*, none of the clinical strains had nonsense mutations in the gene, as observed for *S. mutans* NG5. These results suggested that mutations in the *srtA* gene of laboratory strains Ingbritt and NG5 may be the consequences of long-term subculturing but are not a reflection of variation within the species in nature.

Compared with our previous study that included a small sample [15], more mutation sites were found. All variant sites that had previously been discovered with the smaller sample sizes were identified again in this larger sample, which suggested that certain mutations in the *srtA* gene commonly occur in *S. mutans* strains. The greater number of mutation sites identified in the present study was likely due to the larger number of strains sequenced, which increased the probability of detecting different mutation sites.

In addition to bacterial genetic factors, the results of multiple logistic regression analyses revealed that other factors, including a greater age, prolonged breastfeeding, a high frequency of solid sugar consumption and visible plaque accumulation also contribute to caries in children with *S. mutans*. Older children have more caries because their teeth have been exposed to the environment and risks for a longer time. Although the relationship between prolonged breast-feeding and caries is controversial, prolonged breast-feeding may contribute to dental caries because it allows for the colonization of *S. mutans* [25] and because breast milk is more cariogenic than other types of milk [26]. The consumption of sugars has long been considered a cause of caries because it can be metabolized by *S. mutans* to produce the plaque dextrans essential for acid production [27]. Moreover, children with a higher proportion of VPI were more likely to have high-severity caries. Dental plaque retention increases *S. mutans* colonization such that there is an increase in the risk of caries [13]. This result was in accordance with those of previous studies [2,3].

There are limitations to the present study. The dmft ≥6 set as a cut-off value likely narrowed the selection of candidates for the caries active group. Further study is needed to clarify the mechanism of *srtA* gene mutation and dental caries.

## Conclusions

The present study provided knowledge about the genetic diversity of the *sortase A* gene of *S. mutans* in children with no caries and those with high-severity caries. The results of the study suggested that the locus 168 missense mutation of the *srtA* gene may correlate with caries susceptibility of children with *S. mutans*. In addition, age, duration of breastfeeding, solid sugar consumption, and poor oral hygiene also contributed to this complex disease.

### Availability of supporting data

The nucleotide sequences of the *srtA* gene of *S. mutans* isolates determined in this study have been deposited in the GenBank database under the accession numbers KP301259 - KP301500.

## Abbreviations

*S. mutans*: *Streptococcus mutans*
DNA: Deoxyribonucleic acid
PCR: Polymerase chain reaction
WHO: World Health Organization
VPI: Visible Plaque Index
MSB: Mitis-Salivarius-Bacitracin agar
BHI: brain heart infusion broth
FDI: Federation Dentaire Internationale

## References

[1] Selwitz RH, Ismail AI, Pitts NB (2007). Dental caries. Lancet.

[2] Mulu W, Demilie T, Yimer M, Meshesha K, Abera B (2014). Dental caries and associated factors among primary school children in Bahir Dar city: a cross-sectional study. BMC Res Notes.

[3] Zhou Y, Lin HC, Lo EC, Wong MC (2011). Risk indicators for early childhood caries in 2-year-old children in southern China. Aust Dent J.

[4] Folayan MO, Chukwumah NM, Onyejaka N, Adeniyi AA, Olatosi OO (2014). Appraisal of the national response to the caries epidemic in children in Nigeria. BMC Oral Health.

[5] Struzycka I, Wierzbicka M, Jodkowska E, Rusyan E, Ganowicz E, Fidecki M (2014). Oral health and prophylactic-therapeutic needs of children aged 6 years in Poland in 2012. Przegl Epidemiol.

[6] Qi XQ (2008). Report of the Third National Oral Health Survey in China (In Chinese).

[7] Krzysciak W, Jurczak A, Koscielniak D, Bystrowska B, Skalniak A (2014). The virulence of Streptococcus mutans and the ability to form biofilms. Eur J Clin Microbiol Infect Dis.

[8] Jenkinson HF, Demuth DR (1997). Structure, function and immunogenicity of streptococcal antigen I/II polypeptides. Mol Microbiol.

[9] Lee SF, Boran TL (2003). Roles of sortase in surface expression of the major protein adhesin P1, saliva-induced aggregation and adherence, and cariogenicity of Streptococcus mutans. Infect Immun.

[10] Igarashi T, Asaga E, Goto N (2003). The sortase of Streptococcus mutans mediates cell wall anchoring of a surface protein antigen. Oral Microbiol Immunol.

[11] Lee SF, McGavin MK (2004). Identification of a point mutation resulting in loss of cell wall anchoring activity of SrtA of Streptococcus mutans NG5. Infect Immun.

[12] Napimoga MH, Hofling JF, Klein MI, Kamiya RU, Goncalves RB (2005). Tansmission, diversity and virulence factors of Sreptococcus mutans genotypes. J Oral Sci.

[13] Zhou Y, Yang JY, Zhi QH, Tao Y, Qiu RM, Lin HC (2013). Factors associated with colonization of Streptococcus mutans in 8 to 32-month-old children: a cohort study. Aust Dent J.

[14] Su Z, Fang JQ, Sun ZQ (2008). The sample size calculation and statistical analysis of case control study. Health Statistics (In Chinese).

[15] Zhang XH, Zhou Y, Zhi QH, Tao Y, Lin HC (2012). Genetic polymorphisms of the sortase A gene and early childhood caries in two-year-old children. Arch Oral Biol.

[16] Zhang R, Lin HC, Zhi QH (2008). Caries prevalence of primary teeth and related factors in urban and rural young children of Guangzhou (In Chinese). Chin J Pract Stomatol.

[17] Zhang XH, Zhou Y, Lin HC (2009). Acquisition of Streptococcus mutans and cell adherence among 2-year old children with different caries experience (in Chinese). Chin J Pract Stomatol.

[18] World Health Organization (1997). Oral health survey, Basic Methods.

[19] FDI Commission on Oral Health, Research and Epidemiology (1992). A review of the developmental defects of enamel index (DDE index). Int Dent J.

[20] Ainamo J, Bay I (1975). Problems and proposals for recording gingivitis and plaque. Int Dent J.

[21] Gold OG, Jordan HV, Van Houte J (1973). A selective medium for Streptococcus mutans. Arch Oral Biol.

[22] Shklair IL, Keene HJ (1974). A biochemical scheme for the separation of the five varieties of Streptococcus mutans. Arch Oral Biol.

[23] Martins-Junior PA, Vieira-Andrade RG, Correa-Faria P, Oliveira-Ferreira F, Marques LS, Ramos-Jorge ML (2013). Impact of early childhood caries on the oral health-related quality of life of preschool children and their parents. Caries Res.

[24] Igarashi T (2004). Deletion in sortase gene of Streptococcous mutans Ingbritt. Oral Microbiol Immunol.

[25] Vachirarojpisan T, Shinada K, Kawaguchi Y, Laungwechakan P, Somkote T, Detsomboonrat P (2004). Early childhood caries in children aged 6–19 months. Community Dent Oral Epidemiol.

[26] Bowen WH, Lawrence RA (2005). Comparison of the cariogenicity of cola, honey, cow milk, human milk, and sucrose. Pediatrics.

[27] Moye ZD, Zeng L, Burne RA (2014). Fueling the caries process: carbohydrate metabolism and gene regulation by Streptococcus mutans. J Oral Microbiol.

